# Quality of life after laparoscopic totally extraperitoneal (TEP) inguinal hernia repair with fibrin glue versus tack mesh fixation

**DOI:** 10.1038/s41598-025-26626-5

**Published:** 2025-11-21

**Authors:** Ahmed Anwar, Waleed M. Ghareeb, Omar Yasser, Hamdy Shaban, Sameh T. Abu-Elela

**Affiliations:** General and Gastrointestinal Surgery Department, Faculty of Medicine, Suez Canal University Hospitals, Ismailia, Egypt

**Keywords:** Laparoscopic, Hernia, Glue, TEP, Quality of life, Quality of life, Chronic pain

## Abstract

Mechanical methods are hypothesized to have postoperative pain and more seroma formation than non-mechanical methods due to tissue trauma. Therefore, the current prospective cohort study aimed to assess postoperative pain and quality-of-life (QoL) after fibrin glue versus tack mesh fixation. From July 2022 to December 2023, 80 patients sought TEP at the Suez Canal University Hospitals outpatient clinic. Participants were divided into two groups based on mesh fixation: the Fibrin glue group (FG) and the Tack group (TG). The purpose of this study was to compare the rate of post-operative complications, post-operative pain, length of hospital stays, and wound complications. In the meantime, the two groups QoL were compared using the SF-36 scoring questionnaire and the Caroline Comfort score (CCS). The patients in TG had higher operative time (84.5 ± 5.5 min) compared to patients in FG (78.3 ± 6.4); without statistical significance (*p* = 0.21). The FG had a statistically significant shorter length of hospital stay compared to TG (*p* = 0.02) although the duration till initiation of weight bearing did not have statistical difference between both groups (*p* = 0.09). With a 30-day postoperative follow-up period, overall, there was no difference between both groups regarding the development of postoperative urine retention, seroma or wound infection (*p* = 0.09, 0.32, 0.3; respectively). Furthermore, after 6 months, FG had a higher overall QoL score using both CCS and SF-36 questionnaire (*P* = 0.001 and 0.02; respectively). Glue fixation may have a better quality of life and less postoperative pain; however further clinical trials are still needed.

## Introduction

Inguinal hernias are the most commonly presented abdominal hernias with approximately 20 million people operated on annually throughout the world. They are more commonly reported in young males, manual workers and in low socioeconomic population^1^. The advent of mesh was a breakthrough in the field of hernia surgery. A variety of meshes with variable physical and chemical properties are available for the repair of hernias^2^.Using prosthetic mesh materials, such as nonabsorbable polypropylene used in hernia repair have been known to cause physiological reactions causing pain and heaviness, whereas, recently used lightweight Prolene mesh has decreased pain and groin symptoms^3^. Furthermore, surgical techniques have evolved from open procedures to minimally invasive approaches and hernia repair has followed this same progression. Minimally invasive surgery has advantages of early postoperative recovery, lesser hospital stays, and early return to work. It is also associated with lesser postoperative pain due to smaller incisions with recurrence rates similar to open surgery^4^.A wide variety of mesh fixation techniques are available for laparoscopic hernia repair. These can be broadly divided into mechanical and nonmechanical methods^5^. Mechanical methods include sutures and tissue penetrating fixation devices like tackers. Nonmechanical techniques include self-gripping meshes and tissue adhesives (glues)^1^.

Mechanical methods are hypothesized to cause more postoperative pain and increased risk of seroma formation, hematoma formation, and osteitis pubis due to tissue trauma and also have increased risk of chronic pain due to nerve entrapment. On the other hand, nonmechanical methods do not have these disadvantages^6^.

Chronic pain following inguinal hernia repair has been reported in up to 53% of cases, with approximately 10% of patients experiencing worsened pain postoperatively. This burden is particularly notable in younger patients, those with severe preoperative pain, and in procedures involving heavyweight mesh and open techniques. While fibrin glue presents a promising solution, evidence evaluating its impact on postoperative quality of life remains limited^2,7,8^

Therefore, this study was designed to compare patient-reported outcomes following inguinal hernia repair using fibrin glue versus traditional tack mesh fixation, with a focus on postoperative pain and overall quality of life.

## Patients and methods

### Study design and setting

This is a prospective cohort study on patients who were presenting with uncomplicated inguinal hernia and scheduled for laparoscopic total extraperitoneal (TEP) hernioplasty at Suez Canal University Hospitals in the period of July 2022 to December 2023. Ethical approval for the study was obtained from the Institutional Review Board of Suez Canal University, Faculty of Medicine (code #4846/2022). The study was registered at the www.clinicaltrial.gov under number: NCT06679504.

### Selection criteria and outcome measurement

**Fig. 1:**
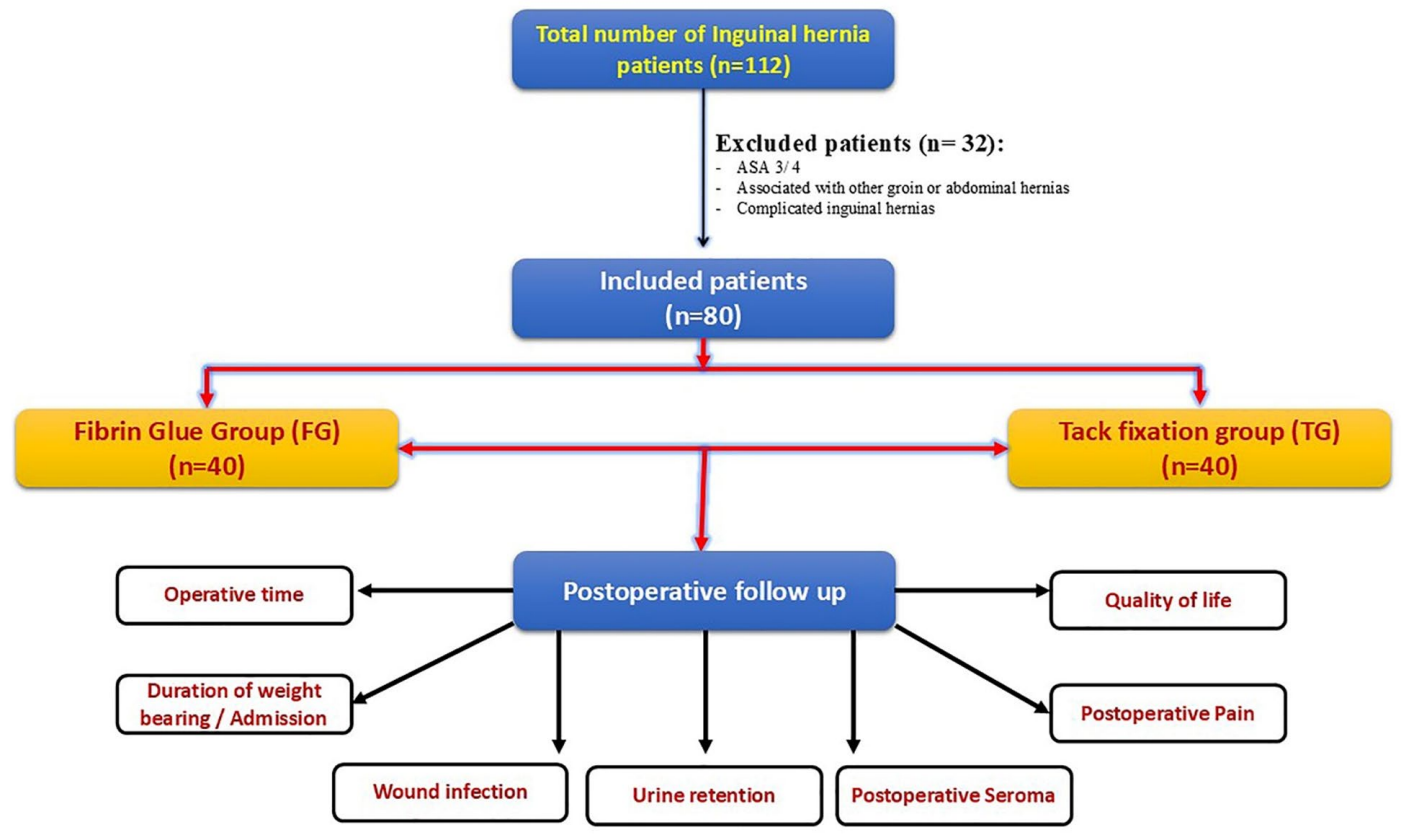
Flow chart depicting the inclusion and exclusion approaches for the current study participants. **ASA**: american society of anesthesiologists.

Male patients underwent laparoscopic TEP repair were divided into Fibrin glue fixation (group A) and Tack fixation (group B). Patients aged between 18 and 60 years old, with inguinal hernia (direct (medial) or indirect (lateral) classified as M3 or L3 depending on the European Hernia society (EHS) classification^9^ and did not undergo hernia repair surgery before were included. According to the guidelines published^10,11^, inguinal hernia repair without fixing the mesh is recommended in almost all hernia types except when defect is larger than 3 cm in medial (direct) hernia [M3 fixation is recommended] and lateral (indirect) hernia [L3 fixation is preferred]. Meanwhile, patients who were unfit for general anesthesia or with American Society of Anesthesiologists (ASA) grade 3 and above, associated with other groin or abdominal hernias, recurrent, or complicated inguinal hernias, were excluded (Fig. 1). In tack mesh fixation, non-absorbable two tackers (one at Cooper’s ligament and another at the medially at the anterior abdominal wall) were used in all patients included in this group.

The main outcome of the present study was to compare quality of life after laparoscopic totally extra peritoneal inguinal hernia repair with fibrin glue versus tack mesh fixation using SF-36 scoring questionnaire^12^ and Carolinas Comfort Scale (CCS)^13^. Furthermore, the postoperative pain has been compared between both groups using the Visual Analogue Scale (VAS) for pain assessment. (0–10, with 0 indicating no pain and 10 indicating the worst possible pain)

### Sample size calculation

The sample size was determined using an online sample size calculator (http://www.raosoft.com/samplesize.html). The proportion of postoperative usage of analgesics among patients operated with laparoscopic TEP and tack fixation vs. fibrin glue fixation was considered 36% and 16%, respectively. According to the previous calculations, 71 patients were required. After accounting for a 10% drop-out, the total required sample size was 80 patients divided upon 2 groups, with the margin of error set at 5% and confidence level of 95%. At the same visiting day at the outpatient clinic, one of the researchers assigned the included patients to one of the study groups in successive manners starting by tack mesh group at a rate of 1:1.

### Statistical analysis

Categorical variables were expressed as frequencies and percentages, while continuous variables were expressed as mean ± standard deviation (SD). Categorical variables were compared using Chi-Square or Fisher’s exact test. Continuous variables were compared using Student t-tests or Mann-Whitney test, as appropriate. All statistical analyses were performed using IBM SPSS software (Version 25.0. Armonk, NY: IBM Corp.). A p-value < 0.05 was considered significant.

## Results

### Characteristics of patients

A total of 80 patients were included in the present study (Fibrin glue group = 40 patients and Tack fixation group = 40 patients). The mean age of patients underwent laparoscopic TEP repair with fibrin glue and tack mesh fixation was 48.95 ± 8.49 and 48.12 ± 11.06 years respectively (*P* = 0.96). Overall, all patients included were males while diabetes was the most common chronic illness among the studied patients without statistical significance between both groups (*P* = 0.2) (Table 1).

**Table 1:** Basic demographic data of the studied group (*N* = 80).

	Fibrin glue group (*n* = 40)	Tack mesh fixation group (*n* = 40)	*P*-value
Age (years), mean ± SD	48.95 ± 8.49	48.12 ± 11.06	0.96
BMI (kg\m2)	24.4 ± 2.3	25.1 ± 3.5	0.302
ASA grade _n, (%)_ I II	34(85%) 6(15%)	33(82.5%) 7(17.5%)	0.98
Gender _n, (%)_ male	40 (100%)	40(100%)	1
Occupation Employee Manual Worker	15(37.5%) 25(62.6%)	13(32.5%) 27(67.5%)	0.87
Marital status Married Single	21(52.5%) 19(47.5%)	25(62.5%) 15(37.5%)	0.69
Smoking status Current smoker Non/ex-smoker	23(57.5%) 17(42.5%)	25(62.5%) 15(37.5%)	0.68
Chronic illness DM HTN IHD	6(15%) 5(10%) 1(0.5%)	3(7%) 3(7%) 0(0%)	0.21
Type of hernia Direct (M3) Indirect (L3)	12(30%) 28(70%)	13(32.5%) 27(67.5%)	0.90
Occurrence of hernia Primary	40(100%)	40(100%)	1

* Statistical significance at *P* < 0.05. BMI: Body Mass Index; DM: Diabetes Mellitus; HTN: Hypertension; IHD: Ischemic Heart Disease; M3: medial/direct hernia with defect > 3 cm; L3: lateral/indirect hernia with defect > 3 cm.

### Comparing the postoperative outcome between fibrin glue vs. tack mesh fixation groups

The patients in tack mesh fixation group had higher operative time (84.5 ± 5.5 min) compared to patients in fibrin glue group (78.3 ± 6.4); however, there was no statistical significance (*p* = 0.21).

Furthermore, the fibrin glue group had statistically significant lower length of hospital stay (1.3 ± 0.3 days) compared to tack mesh group (1.8 ± 0.8 days) (*p* = 0.02) although the duration till initiation of weight bearing did not have statistical difference between both groups (2.28 ± 0.9 vs. 2.38 ± 1.3 days, respectively *p* = 0.09).

With following up patients for 30 days postoperative, Overall, there was no difference between both groups regarding the development of urinary retention, seroma or wound infection. (Table 2)

**Table 2:** Postoperative data of the studied group (*N* = 80).

	Fibrin glue group (*n* = 40)	Tack mesh fixation group (*n* = 40)	*P*-value
Operative time _(min)_	78.3 ± 6.4 [95%CI = 76.3–80.3]	84.5 ± 5.5 [95%CI = 82.8–86.2]	0.21
Duration till initiation of weight bearing _(days)_	2.28 ± 0.9 [95%CI = 2–2.6]	2.38 ± 1.3 [95%CI = 1.9–2.8]	0.09
Wound infection	1(2.5%)	2(5%)	0.3
Urinary retention	3(7.5%)	7(17.5%)	0.09
Seroma	2(5%)	3(7.5%)	0.32
Length of postoperative hospital stay (days)	1.3 ± 0.3 [95%CI = 1.2–1.4]	1.8 ± 0.8 [95%CI = 1.6–2.1]	**0.02** ^*^

* Statistical significance at *P* < 0.05.

With further follow up of the patients 6 months after surgery, for postoperative pain assessment, the patients in fibrin glue group had significantly lower visual analogue score (VAS) (1.48 ± 0.5) compared to patients in tack mesh fixation (5.7 ± 1.36) (*p* < 0.001). Furthermore, the fibrin glue group had a statistically significant higher overall quality of life score compared to tack mesh fixation group using both CCS and SF-36 questionnaire (*P* = 0.001 and 0.02; respectively) (Table 3).

**Table 3:** Postoperative pain and quality of life assessment (*N* = 80).

	Fibrin glue group (*n* = 40)	Tack mesh fixation group (*n* = 40)	*P*-value
VAS score	1.48 ± 0.51 [95%CI = 1.32–1.64]	1.95 ± 0.82 [95%CI = 1.69–2.2]	**0.002** ^*^
**CCS score**, ***Mean*** *± SD*	99 ± 7 [95%CI = 96.8–101.2]	105 ± 6 [95%CI = 103.1–106.9]	**0.001** ^*^
SF-36 Questionnaire score (%), ***Mean*** *± SD*	79 ± 11 [95%CI = 75.6–82.4]	71 ± 17 [95%CI = 65.7–76.3]	**0.02** ^*^

* Statistical significance at *P* < 0.05. CI: Confidence interval; VAS: Visual Analogue Scale; CCS: Caroline Comfort Score; SD: Standard deviation.

## Discussion

The advent of laparoscopy brought new surgical approaches to inguinal hernia repair (IHR), and rigorous analysis comparing laparoscopic techniques to open has determined that the laparoscopic approach offers less postoperative pain, faster return to daily activities, and overall fewer complications^14^. Moreover, when compared to mechanical fixation procedures, fibrin glue mesh fixation has been linked to a reduced incidence of persistent postoperative inguinal discomfort and hematoma development after laparoscopic inguinal hernia repair, while retaining comparable recurrence rates^15,16^. This study aimed at decreasing morbidity and improving the quality of life of inguinal hernia patients through mesh fixation by fibrin glue instead of tack fixation. The present study participants were monitored for up to 30 days following surgery. The VAS, which measures pain, revealed a significant difference favoring the fibrin glue group (*P* = 0.002). As a result, the fibrin glue group outperformed the Tack mesh fixation group in terms of quality of life as measured by the CCS and SF-36 questionnaires (*P* = 0.001 and 0.02, respectively). Despite the fact that mesh hernioplasty has significantly decreased the likelihood of hernia recurrence, it is linked to the morbidity of chronic postoperative discomfort, which can significantly lower quality of life. Chronic discomfort after hernia repair has been attributed to a number of different etiologies.

In a recent meta-analysis, including 2312 patients from 18 randomized clinical trials (RCTs), Kitching et al. has reported that mesh fixation using fibrin glue has demonstrated superiority over tackers in minimizing postoperative pain and reducing the incidence of hematoma formation. These advantages contribute to faster recovery and earlier return to daily activities or work. Surgeons should consider these benefits during the informed consent process for laparoscopic inguinal and femoral hernia repair^17^. Several mechanisms have been proposed to explain the development of postoperative pain following tack mesh fixation. One avoidable cause of postoperative pain following tack mesh fixation is nerve entrapment during mesh placement, which can lead to neuropathic symptoms. Additionally, meshoma formation, a localized mesh-induced fibrosis, may incorporate adjacent nerves, contributing to persistent discomfort. It has also been hypothesized that mesh contraction over time may irritate surrounding nerve endings, exacerbating pain. In some patients, chronic inflammation triggered by the foreign body response to the mesh material further contributes to long-term discomfort and impaired quality of life^18,19^.

The severity of preoperative symptoms as assessed by the modified CCS is a strong predictor of reported postoperative complaints and is negatively correlated with the size of a direct defect, if one occurs. A CCS-based questionnaire specifically designed for hernias is being used for inguinal hernia repair in the preoperative and early postoperative stages for the first time, as far as we are aware. Of the 320 patients whose postoperative quality of life was assessed using the CCS comprehensive scoring technique, 55 and 38.1% were scored as “very satisfied” or “satisfied” at their initial visit, according to Berney et al. The patients’ satisfaction level rose even further at the second (6-week) visit, hitting 86.5 and 12.7%, respectively. The median and average scores for every group. Even though patients 50 years of age or younger had higher CCS scores than patients over 50, and the bilateral surgery group had higher CCS scores than the unilateral surgery group at both 2 and 6 weeks, the average and median scores in each group and at both time points were at least in the “satisfied” to “very satisfied” range^20^. This agrees with the results of a meta-analysis indicate a statistically significant reduction in chronic pain with the use of tissue glue fixation (OR 0.40; 95% CI 0.21–0.76; *p* = 0.005)^21^. Furthermore, findings were consistent with other studies, which demonstrated reduction in hypoesthesia in the tissue glue arm over the spiral tack arm (*p* = 0.045). However, another study did not show a difference in chronic pain in the ilioinguinal and genitofemoral areas using a VAS (*p* = 0.619)^22^.

Regarding operative data, we found that hospital stay, follow up time and duration till initiation of weigh bearing were shorter among fibrin glue group but with statistical insignificant differences (*p* > 0.05). When compared to the tacker group, the fibrin glue fixation group hospital stay lasted noticeably less. This can be explained by the fibrin glue group experiencing reduced discomfort and early patient mobilization. While the difference in weight-bearing initiation time did not reach statistical significance, this does not preclude its clinical relevance. Even modest improvements in early mobilization can impact patient comfort, rehabilitation speed, and discharge planning—especially in high-volume surgical settings. Therefore, interpreting outcomes should balance statistical metrics with practical, patient-centered implications.

Numerous trials comparing fibrin glue and tackers for mesh fixation revealed that the fibrin glue group required far less postoperative analgesics. Studies comparing fibrin glue fixation and sutures even in open hernia surgery showed that the fibrin glue group experienced less postoperative discomfort^23,24^.

Regarding the emergence of seroma or urine retention, there was no difference between the two groups, although it’s possible that the tackers produced more trauma than fibrin glue. A propensity score matched studies revealed no discernible difference between the fibrin glue and tacker groups in terms of postoperative problems such as hematoma, seroma, and urine retention According to some other researchers, however, the incidence of postoperative urinary retention was considerably higher in the tacker group than in the fibrin glue group, and it was connected with the quantity of tackers utilized^23,25,26^.

The current study identified some gaps in the body of knowledge on this subject, which may open the door for more research. More prospective randomized studies on the topic are required, ideally with controls for sex and age. It is necessary to examine if using varying amounts of fibrin glue causes any variations in long-term recurrence. Moreover, further researches comparing the absorbable tacker to other nonabsorbable fixing devices are also required.

## Conclusion

Glue fixation may be effective in reducing the incidence of pain without increasing the risk of recurrence. Future research should consider both the effectiveness and cost-effectiveness of fixation techniques alongside the type of mesh and the size and location of the hernia defect.

## Data Availability

All data are available with the corresponding author on reasonable request.

## References

[1] Nizam, S. et al. Mesh fixation with fibrin glue versus tacker in laparoscopic totally extraperitoneal inguinal hernia repair. *ANZ J. Surg.* **91** (10), 2086–2090 (2021). 34448342 10.1111/ans.17165

[2] Ladurner, R. et al. Long term outcome and quality of life after open incisional hernia repair–light versus heavy weight meshes. *BMC Surg.* **11**, 25 (2011). 21917180 10.1186/1471-2482-11-25 PMC3180243

[3] Shankar, D. A. et al. Factors associated with Long-term outcomes of umbilical hernia repair. *JAMA Surg.* **152** (5), 461–466 (2017). 28122076 10.1001/jamasurg.2016.5052 PMC5831449

[4] Memon, G. A., Shah, S. K. A., Habib, R. & Ur An experience with mesh versus darn repair in inguinal hernias. *Pak J. Med. Sci.* **33** (3), 699–702 (2017). 28811798 10.12669/pjms.333.13257 PMC5510130

[5] Koju, R. et al. Transabdominal Pre-peritoneal mesh repair versus lichtenstein’s hernioplasty. *J. Nepal. Health Res. Counc.* **15** (2), 135–140 (2017). 29016583 10.3126/jnhrc.v15i2.18202

[6] Iftikhar, N. & Kerawala, A. Quality of Life After Inguinal Hernia Repair. *Pol. Przegl Chir.* **93** (3), 1–5 (2021). 33949330 10.5604/01.3001.0014.8218

[7] Jensen, K.K., . Recovery after abdominal wall reconstruction. Dan med J,** 64** (3), B5349 (2017). 28260602

[8] Sadeghi, M. M. Comparison of single absorbable tacker vs. conventional method in fixating the mesh in bilateral inguinal hernia undergoing laparoscopic transabdominal preperitoneal (TAPP): A randomized control trial study. *J. Res. Med. Sci.* **29**, 25 (2024). 38855564 10.4103/jrms.jrms_161_23 PMC11162083

[9] Miserez, M. et al. The European hernia society groin hernia classification: simple and easy to remember. *Hernia* **11** (2), 113–116 (2007). 17353992 10.1007/s10029-007-0198-3

[10] Bittner, R. et al. *Guidelines for laparoscopic (TAPP) and endoscopic (TEP) treatment of inguinal hernia [International Endohernia Society (IEHS)].* Surgical endoscopy, **25**(9): 2773–2843. (2011). 10.1007/s00464-011-1799-6 PMC3160575 21751060

[11] Mayer, F. et al. When is mesh fixation in TAPP-repair of primary inguinal hernia repair necessary? The register-based analysis of 11,230 cases. *Surg. Endosc*. **30** (10), 4363–4371 (2016). 26886454 10.1007/s00464-016-4754-8 PMC5009149

[12] Lins, L. & Carvalho, F. M. SF-36 total score as a single measure of health-related quality of life: scoping review. *SAGE open. Med.* **4**, 2050312116671725–2050312116671725 (2016). 27757230 10.1177/2050312116671725 PMC5052926

[13] Heniford, B. T. et al. Carolinas comfort scale as a measure of hernia repair quality of life: A reappraisal utilizing 3788 international patients. *Ann. Surg.* **267** (1), 171–176 (2018). 27655239 10.1097/SLA.0000000000002027

[14] Patel, L.Y., Lapin, B., Gitelis, M.E., Brown, C., Linn, J.G., Haggerty, S., Denham, W., Butt, Z., Barrera, E., Joehl, R. and Carbray, J. Long-term patterns and predictors of pain following laparoscopic inguinal hernia repair: a patient-centered analysis. *Surgical endoscopy*, **31**(5), 2109-2121.(2017). 10.1007/s00464-016-5207-0 27585467

[15] Habib Bedwani, N. A. R. et al. Glue versus mechanical mesh fixation in laparoscopic inguinal hernia repair: meta-analysis and trial sequential analysis of randomized clinical trials. *Br. J. Surg.* **108** (1), 14–23 (2021). 33640918 10.1093/bjs/znaa002

[16] Hu, N. et al. Efficacy and safety of glue mesh fixation for laparoscopic inguinal hernia: A meta-analysis of randomized controlled trials. *Asian J. Surg.* **46** (9), 3417–3425 (2023). 37037745 10.1016/j.asjsur.2023.03.146

[17] Kitching, S. et al. Glue versus tackers for mesh fixation in laparoscopic inguinal hernia repair: a meta-analysis and trial sequential analysis. *Hernia* **29** (1), 134 (2025). 40186768 10.1007/s10029-025-03315-w PMC11972183

[18] Friis-Andersen, H. Letter to the editor on absorbable versus non-absorbable tacks for mesh fixation in laparoscopic ventral hernia repair: A systematic review and meta-analysis [Int. J. Surg. 53 (2018) 184–192]. *Int. J. Surg.* **82**, 54–55 (2020). 32750490 10.1016/j.ijsu.2020.07.045

[19] Fortelny, R. H. Fixation techniques in inguinal hernia repair, what is really new? *Hernia* **24** (1), 209–211 (2020). 31559503 10.1007/s10029-019-02052-1

[20] Berney, C. R. & Descallar, J. Review of 1000 fibrin glue mesh fixation during endoscopic totally extraperitoneal (TEP) inguinal hernia repair. *Surg. Endosc*. **30** (10), 4544–4552 (2016). 26895903 10.1007/s00464-016-4791-3

[21] Shah, N. S. et al. Mesh fixation at laparoscopic inguinal hernia repair: a meta-analysis comparing tissue glue and tack fixation. *World J. Surg.* **38** (10), 2558–2570 (2014). 24770891 10.1007/s00268-014-2547-6

[22] Wikiel, K. J. & Eid, G. M. Groin defects seen at extra-peritoneal laparoscopic dissection during surgical treatment of athletic pubalgia. *Surg. Endosc*. **29** (7), 1695–1699 (2015). 25294545 10.1007/s00464-014-3866-2

[23] Chung, Y. et al. Feasibility of totally extraperitoneal (TEP) laparoscopic hernia repair in elderly patients. *Hernia* **23** (2), 299–303 (2019). 30511101 10.1007/s10029-018-1869-y

[24] de Goede, B. et al. Meta-analysis of glue versus sutured mesh fixation for Lichtenstein inguinal hernia repair. *Br. J. Surg.* **100** (6), 735–742 (2013). 23436683 10.1002/bjs.9072

[25] Chandra, P., Phalgune, D. & Shah, S. Comparison of the clinical outcome and complications in laparoscopic hernia repair of inguinal hernia with mesh fixation using fibrin glue vs tacker. *Indian J. Surg.* **78** (6), 464–470 (2016). 28100943 10.1007/s12262-015-1410-9 PMC5218932

[26] Kaul, A. et al. Staple versus fibrin glue fixation in laparoscopic total extraperitoneal repair of inguinal hernia: a systematic review and meta-analysis. *Surg. Endosc*. **26** (5), 1269–1278 (2012). 22350225 10.1007/s00464-011-2025-2

